# SARS-CoV-2 Infection: What Is Currently Known about Homocysteine Involvement?

**DOI:** 10.3390/diagnostics13010010

**Published:** 2022-12-21

**Authors:** Nina Filip, Elena Cojocaru, Oana Viola Badulescu, Andreea Clim, Alin Constantin Pinzariu, Gabriela Bordeianu, Alina Elena Jehac, Cristina Elena Iancu, Cristiana Filip, Minela Aida Maranduca, Ivona Andreea Sova, Ionela Lacramioara Serban

**Affiliations:** 1 Department of Morpho-Functional Sciences (II), Discipline of Biochemistry, Faculty of Medicine, “Grigore T. Popa” University of Medicine and Pharmacy, 700115 Iasi, Romania; 2 Department of Morpho-Functional Sciences (I), Discipline of Morphopathology, Faculty of Medicine, “Grigore T. Popa” University of Medicine and Pharmacy, 700115 Iasi, Romania; 3 Department of Morpho-Functional Sciences (II), Discipline of Pathophysiology, Faculty of Medicine, “Grigore T. Popa” University of Medicine and Pharmacy, 700115 Iasi, Romania; 4 Department of Morpho-Functional Sciences (II), Discipline of Physiology, Faculty of Medicine, “Grigore T. Popa” University of Medicine and Pharmacy, 700115 Iasi, Romania; 5 Department of Dentoalveolar and Maxillofacial Surgery, Faculty of Dental Medicine, “Grigore T. Popa” University of Medicine and Pharmacy, 700115 Iasi, Romania; 6 Department of Biochemistry, Faculty of Pharmacy, “Grigore T. Popa” University of Medicine and Pharmacy, 700115 Iasi, Romania; 7 IOSUD Faculty of Medicine, “Grigore T. Popa” University of Medicine and Pharmacy, 700115 Iasi, Romania

**Keywords:** homocysteine, SARS-CoV-2, thrombosis, cardiovascular disease, MTHRF

## Abstract

Since December 2019, severe acute respiratory syndrome coronavirus 2 (SARS-CoV-2) has spread rapidly throughout the world causing health, social and economic instability. The severity and prognosis of patients with SARS-CoV-2 infection are associated with the presence of comorbidities such as cardiovascular disease, hypertension, chronic lung disease, cerebrovascular disease, diabetes, chronic kidney disease, and malignancy. Thrombosis is one of the most serious complications that can occur in patients with COVID-19. Homocysteine is a non-proteinogenic α-amino acid considered a potential marker of thrombotic diseases. Our review aims to provide an updated analysis of the data on the involvement of homocysteine in COVID-19 to highlight the correlation of this amino acid with disease severity and the possible mechanisms by which it intervenes.

## 1. Introduction

The World Health Organization (WHO) declared in early 2020 that severe acute respiratory syndrome coronavirus 2 (SARS-CoV-2) infection was a public health emergency of international concern. To date, considerable efforts have been made to both prevent and diagnose and treat SARS-CoV-2 infection. SARS-CoV-2 enters host cells via the angiotensin-converting enzyme 2 (ACE2) receptor and can infect the heart, vascular tissues, and circulating cells [1,2]. 

Pneumonia, multiorgan failure, or even death was highlighted in patients with SARS-CoV-2 infection. In order to manage and treat patients with Coronavirus disease 2019 (COVID-19), it is really important to know the risk factors (comorbidities) and specific biomarkers. Hypertension, cardiovascular diseases, diabetes, kidney diseases, lung diseases, and cancer were the comorbidities associated with the severity of COVID-19 [3,4,5,6]. Laboratory biomarkers of organ damage have an important role in the diagnosis and prognosis of patients infected with SARS-CoV-2 because the virus has been identified in endothelial, liver, kidney, lung and neuronal cells [7,8,9,10]. Proinflammatory cytokines, neuron-specific enolase, lactate dehydrogenase, aspartate transaminase, neutrophil count, neutrophil-lymphocyte ratio, troponins, creatine kinase, myoglobin, D-dimer, brain natriuretic peptide and its N-terminal prohormone are the most widely used biomarkers to predict disease severity [11,12,13].

Thrombosis is one of the most serious complications that can occur in patients infected with SARS-CoV-2 [14,15,16,17]. Helms et al. [18] evaluated the thrombotic risk in a number of 150 patients with COVID-19 and reported a percentage of 42% with thrombotic complications, mainly pulmonary embolisms. It is clear that patients with SARS-CoV-2 may have coagulation abnormalities leading to a hypercoagulability state.

**Figure 1:**
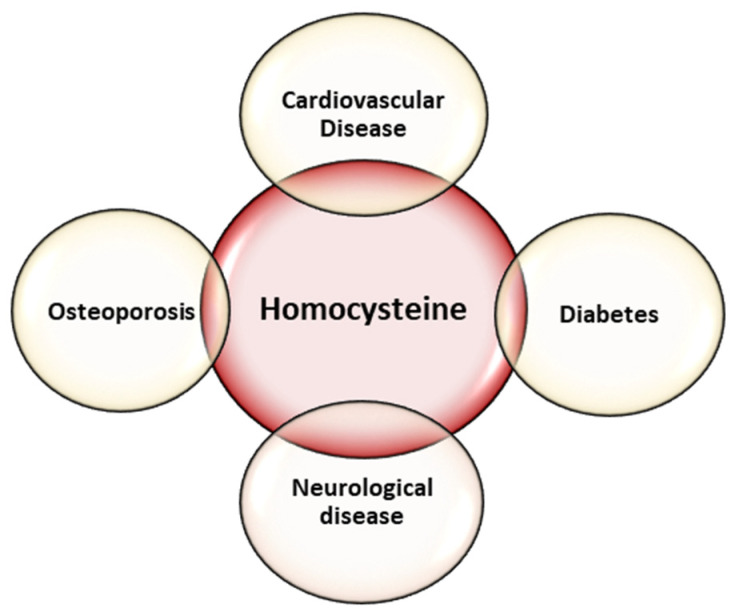
Diseases associated with homocysteine.

Homocysteine is a non-proteogenic α-amino acid that is formed in the metabolism of methionine. The increase in serum homocysteine values can be caused either by the excessive intake of methionine, or most frequently, by the blocking of one of the metabolic pathways through dietary deficiency of folic acid, vitamin B12 and vitamin B6. Elevated serum homocysteine concentration is thought to be involved in many diseases, including neurological diseases [19,20,21], cardiovascular diseases [22,23,24], osteoporosis [25,26], and diabetes [27] (Figure 1).

Numerous studies indicate that elevated serum homocysteine levels have toxic effects on the vascular endothelium, causing cellular dysfunction [27,28,29,30,31,32,33]. Damage to the vascular endothelium leads to thrombus formation, generating a hypercoagulability state.

Considering the previously mentioned data on the state of hypercoagulability in patients with SARS-CoV-2 infection and that homocysteine is a marker of thrombotic diseases, we aimed to review the available data on the involvement of hyperhomocysteinemia (HHCY) in the prognosis of patients infected with this virus.

## 2. Homocysteine Metabolism

**Figure 2:**
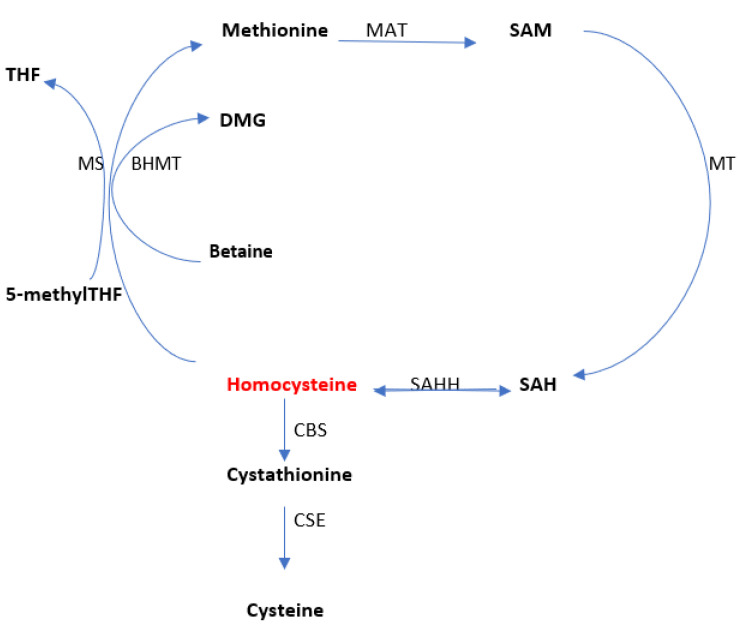
The overview of homocysteine metabolism. Methionine adenosyltransferase (MAT); methyltransferase (MT); S-adenosylhomocysteine hydrolase (SAHH); betaine-homocysteine S-methyl transferase (BHMT); Cystathionine β-synthase (CBS); Cystathionine γ-lyase (CSE); methionine synthase (MS). Adapted from Moretti et al. [34].

Homocysteine is a sulfated amino acid, formed during the metabolism of methionine, an essential amino acid present in proteins of animal origin [34]. Homocysteine is metabolized in two ways, namely: remethylation (through which methionine is regenerated) and transsulfuration (through which it degrades to cysteine) (Figure 2). The serum level varies according to the two metabolic pathways.

Methionine is the body’s main donor of methyl radicals in the form of S-adenosylmethionine (SAM) which is then converted into S-adenosylhomocysteine (SAH). Homocysteine is generated by the cleavage of SAH, the reaction catalyzed by S-adenosylhomocysteine hydrolase (SAHH; EC 3.3.1.1). Once synthesized, homocysteine quickly undergoes remethylation to methionine in a reaction catalyzed by methionine synthase (MS; EC 2.1.1.13) (which uses N5-methyltetrahydrofolate as a methyl donor and cobalamin as the cofactor). N5-methyltetrahydrofolate is formed by the reduction of N5,10-methylenetetrahydrofolate in a reaction catalyzed by N5,10-methylenetetrahydrofolate reductase (MTHFR; EC 1.5.1.20) [35,36,37].

Remethylation of homocysteine is also achieved via betaine (derived from choline) under the action of betaine-homocysteine S-methyl transferase (BHMT; EC 2.1.1.5), betaine being then converted to dimethylglycine. This last reaction occurs only in the liver and kidneys, while remethylation via methionine synthase is distributed in all tissues [38,39].

Methylcobalamin receives the methyl group from S-adenosylmethionine (SAM) or 5-methyltetrahydrofolate (5-methylTHF), the active form of folic acid. After remethylation, methionine can be reused to produce SAM, the body’s “universal methyl group donor,” which actively participates in numerous metabolic pathways that include myelin methylation, the synthesis of carnitine, coenzyme Q10, creatine, epinephrine, melatonin, methylcobalamin, and phosphatidylcholine [40,41,42]. 

Research has shown that the accumulation of high amounts of homocysteine and adenosine at the cellular level causes all methylation reactions to be completely inhibited [43,44,45]. Homocysteine is converted to cysteine via cystathionine. Cystathionine β-synthase (CBS; EC 4.2.1.22) catalyzes the first step of transsulfuration and allows the condensation of homocysteine and serine to generate cystathionine. It uses vitamin B6 in its active form (pyridoxal 5-phosphate) as a co-factor. Cystathionine γ-lyase (CSE; EC 4.4.1.1) catalyzes the hydrolysis of cystathionine to form α-ketobutyrate and cysteine, and is also dependent on pyridoxal-5-phosphate [46,47].

The amino acids cysteine and taurine are important compounds at the cardiac level, and for liver detoxification, cholesterol excretion, bile salt formation and glutathione production [48,49]. Recent studies mention that 5-methylTHF, methylcobalamin, betaine, pyridoxal 5-phosphate, and N-acetylcysteine significantly lower elevated homocysteine levels [50,51,52].

## 3. Hyperhomocysteinemia and Vascular Damage

### 3.1. Overview of Hyperhomocysteinemia

In plasma, homocysteine can be found bound to proteins, but also in free, oxidized, or disulfide forms [53]. Plasma homocysteine concentrations of less than 15 μM are considered normal [54,55]. The increase in plasma homocysteine concentration above the normal values is defined as hyperhomocysteinemia. Depending on the concentration of homocysteine, it can be of several types, namely: moderate hyperhomocysteinemia (15–30 μM), intermediate hyperhomocysteinemia (30–100 μM) and severe hyperhomocysteinemia (>100 μM) [56].

The increase in the plasma level of homocysteine can be due to genetic, nutritional, or pathological factors (Table 1).

Changes in homocysteine plasma levels are directly related to the genetic background.

The activity of enzymes involved in homocysteine metabolism is modulated by variants of the genes that encode these enzymes. A C677T point mutation in the gene encoding MTHFR is the most common genetic cause of HHCY. Homozygous carriers may have a moderate increase in homocysteine levels and may experience varying degrees of symptoms caused by venous or arterial thrombosis [79,80].

Another mutation in the MTHFR gene, polymorphism 1298A > C (p. E429A) is associated with decreased MTHFR activity that is more pronounced in the homozygous compared with the heterozygous state, leading to HHCY [81]. Another enzyme involved in the homocysteine remethylation pathway is methionine synthase (MS). The MTR 2756A > G (p. D919G) and MTRR 66A > G polymorphisms can affect the levels of homocysteine [82]. In addition to genetic factors, HHCY can be attributed to a deficiency of vitamins B6, B12 or folate, which plays a crucial role in the homocysteine remethylation pathway, without which, homocysteine would fail [83]. The prevalence of HHCY can vary significantly between populations and, in addition to the previously discussed factors, also depends on age, sex, alcoholism, smoking and medication [28,46].

Since the 1950s, it has been highlighted through numerous studies that megaloblastic anemias caused by folate or vitamin B12 deficiencies can be associated with thromboembolic accidents [84]. It has been shown that there is a directly proportional relationship between folic acid deficiency and elevated homocysteine levels in the risk of coronary heart disease. Low plasma concentration of folic acid is associated with an increased risk of fatal coronary heart disease and an increase in plasma homocysteine [85]. Epidemiological data have shown that homocysteine can be considered a coronary risk factor because homocysteine plasma concentrations are constantly increased in patients with cardiovascular diseases compared to normal subjects.

The coenzymes pyridoxal phosphate, methyl-tetrahydrofolate and cobalamin have an essential role for the enzymes involved in the metabolism of homocysteine and are dependent on the consumption of vitamin B6, vitamin B9 and vitamin B12, respectively. There is a non-linear and inversely proportional association between homocysteine plasma concentration and folate level as well as vitamin intake. Likewise, weaker inversely proportional associations were observed between the concentration of vitamin B12 and plasma pyridoxal phosphate, with the intake of B6, but not with the intake of vitamin B12. It has been shown that a diet rich in cereals and folic acid derivatives contributes to an increase in folate plasma levels and implicitly a decrease in homocysteine concentration [86]. In patients infected with Helicobacter pylori, a deficient absorption of vitamin B6, B12 and acid-causing HHCY have been noted. According to Selhub [87], the prevalence of HHCY would reach an average of 30% among subjects aged between 65 and 80 years and 40–60% in patients over 80 years of age. This increased frequency of HHCY among the geriatric population was explained by a deficiency in B vitamins, estimated at 29% in the elderly and 55% in the very old [88]. It should be noted that in elderly people, folic acid supplementation must be systematically associated with vitamin B12, because it is shown that strong doses of folic acid can lead to a deterioration of cognitive functions, masking the possible deficiency in vitamin B12 [89]. Men have a higher level of homocysteine than women, due to the muscle mass that is better developed and the effects of sex hormones, a fact that was confirmed by a study conducted on a group of people of both sexes, male and female. [90]. Part of the relationship between women’s age and HHCY could be explained by the onset of menopause. In females, an increase in homocysteine is observed after menopause [91].

### 3.2. Hyperhomocysteinemia and Endothelial Dysfunction

Alterations in the balance between vasodilators and vasoconstrictors produced by the endothelium are defined as endothelial dysfunction, associated with atherosclerosis and cardiovascular diseases [92]. High homocysteine levels have toxic effects on the vascular endothelium, damaging it and causing cellular dysfunction, followed by platelet activation, and thrombus formation, thus creating a hypercoagulable state [93]. Regarding venous thromboembolic disease, an increase in the risk of venous thrombosis of 60% (retrospective studies) and 27% (prospective studies) was found for an increase in plasma homocysteine level of 5 mmol/L. The same study showed a 20% increased risk of venous thrombosis in subjects carrying the MTHFR677TT genotype compared to patients with the MTHFR677CC genotype. Therefore, this study incriminates homocysteine as a risk factor for venous thromboembolic disease [94]. Several mechanisms have been proposed through which homocysteine affects the function of the vascular endothelium, among which we mention: increasing oxidative stress, limiting the bioavailability of nitric oxide, stimulating smooth cell proliferation and changing the properties of the elastic wall [95]. Oxidative inactivation of nitric oxide, an important vasodilator, can be a mechanism for endothelial dysfunction in HHCY [96]. The loss of endothelium-mediated vasodilator ability leads to the tilting of the vascular balance towards an abnormally constrictive inflammatory prothrombotic state and is considered to be one of the most rapid manifestations of cardiovascular damage and precedes the formation of atherosclerotic plaques [92]. More research is needed to better understand the exact mechanism by which homocysteine affects endothelial function.

### 3.3. Hyperhomocysteinemia and Cardiovascular Disease

Cardiovascular disease (CVD) is the leading global cause of mortality, with thrombotic complications playing a major role [97]. One of the most incriminated mechanisms of CVD is the atherosclerotic process, in which lipids accumulate at the subendothelial level, resulting in a low-grade inflammatory lesion and transforming the endothelium into a phenotype prone to inflammation and thrombosis. Two hypotheses have been formulated to explain the atherogenicity of the increase in the plasma level of homocysteine: the lipid hypothesis, according to which the alteration of lipoprotein metabolism secondarily induces a touch of the vascular wall, and the inflammatory hypothesis dominated by the direct aggression of cells and vascular tissue.

Lipid peroxidation of low-density lipoproteins (LDL) induces fragmentation of apolipoproteins (apo) B100 initially for their capture by macrophage scavenger receptors. This phenomenon would be responsible to a certain extent for atherogenicity. Sulfated derivatives possessing a free thiol group are likely to cause the oxidation of LDL by endothelial cells and macrophages in vitro [98]. McCully [99] developed the hypothesis that homocysteine would be the main sulfate compound favoring the oxidation of LDL in vivo, a property of its vascular pathogenicity. The ability of homocysteine to modify the physicochemical and biological properties of LDL in vitro has been confirmed. Incubation of LDL in the presence of homocysteine induces a decrease in their content in polyunsaturated fatty acids, the formation of terminal substances of lipid peroxidation (substances that react to thiobarbituric acid) and the fragmentation of apolipoproteins B100 [100]. On the other hand, the thiolation of LDL by homocysteine confers certain pro-atherogenic properties similar to oxidized LDL [101]. Additionally, clinical trial results support a relationship between HHCY and lipid peroxidation in humans [102]. Therefore, the existence of a positive correlation between homocysteinemia and the plasma concentration of a new marker of lipid peroxidation, F2-isoprostane, was reported [103]. 

In contrast, other studies have provided hypotheses going against the lipid hypothesis. Indeed, the oxidation susceptibility of LDL from patients homozygous for CBS deficiency is similar to that of LDL from control subjects [104]. In vitro, homocysteine concentrations between 25 and 500 nmol/L would protect LDL against oxidation, arguing in favor of a paradoxical antioxidant effect of homocysteine [105].

In conclusion, most of the in vitro studies prove that homocysteine induces structural changes at the LDL level, but most of the in vivo results do not confirm the hypothesis that the atherogenicity of HHCY could be related to its ability to oxidize lipoproteins. These studies do not even allow to disprove the lipid hypothesis since first of all the existence of circulating oxidized LDL is controversial, the degree of oxidation of LDL is evaluated by determining their content in thiobarbituric acid which does not allow to highlight the low variations due to its vast variability. Another reason is that the LDL of homozygous CBS-deficient patients is not more susceptible to oxidation in vitro than control subjects [106].

The atherogenicity of HHCY could also result from a primitive activity of vascular endothelial cells leading to their functional dysregulation, followed by platelet and leukocyte activation and proliferation of smooth muscle cells simultaneously with the modification of the sub-endothelial matrix [44,45].

The deleterious action of homocysteine on endothelial cell function has been demonstrated both in vitro and in vivo. In vitro, non-toxic concentrations (less than 1mmol/l) of homocysteine alter the production or activation by endothelial cells of mediators involved in platelet aggregation processes, coagulation and fibrinolysis [107]. They alter the production and bioavailability of endothelial vasodilators and vasoconstrictor mediators.

These experimental results were confirmed by clinical studies indicating that HHCY is accompanied by variations in the plasma concentration and activity of certain endothelium-derived mediators: increased concentrations of von Willebrand factor, endothelin-1 and adhesion molecules such as ICAM-1 (intercellular adhesion molecule-1) and VCAM-1 (vascular cell adhesion molecule-1), decreasing the activity of antithrombin III [108]. At the same time, HHCY is accompanied by alteration of endothelium-dependent vasodilatation, without alteration of endothelium-independent vasodilatation. The pro-atherogenic action of HHCY could come from altering the functionality of the vascular endothelium, causing the induction of a procoagulant and proinflammatory state as well as the deregulation of vascular tone.

The mitogenic action of homocysteine on smooth muscle cells and its toxicity on vascular connective tissue was revealed by histological analysis of the arteries of homocystinuric patients, then confirmed experimentally in animals and in vitro [109]. The mitogenic action would be directly related to the induction of the expression of the regulatory cyclins D1 and A as well as the activation of the transcription factor NF-κB in smooth muscle cells [110]. This action would be indirectly the result of the previously evoked endothelial lesions, as well as the associated thrombotic processes, causing the intraparietal release of platelet factors such as PDGF (platelet-derived growth factor). The aggression of the vascular connective tissue by homocysteine could achieve multiple mechanisms. These include stimulation of collagen synthesis by smooth muscle cells, inhibition of intrachain bond formation between primitive collagen and elastin molecules, induction of collagenases, acceleration of elastin fiber degradation, and induction of cellular secretion of insoluble proteoglycans due to their exclusively sulfated character.

The lipid and inflammatory hypotheses come together to explain the deleterious effects of HHCY. In fact, modified (by homocysteine) low-density lipoproteins (LDL) exhibit biological properties that lead to a dysregulation of the functional state of endothelial cells, comparable to that described for the direct effect of homocysteine on the vascular endothelium. Free radicals originating from the autoxidation of homocysteine or by deregulation of the thiol-redox status, interact with LDL, oxidizing them, which potentiates the harmful effects on the arterial wall. The pathophysiology of the homocysteine-vascular wall interaction is complex and multifactorial.

Since the identification of the main “traditional” risk factors (diabetes mellitus, hypertension, dyslipidemia) for CVD, more than 100 “novel” risk factors have been proposed and studied as potential causative agents, triggers or therapeutic targets [111]. Homocysteine represents one of these “novel” markers that have generated significant interest. Severe HHCY has been observed in individuals with homozygous MTHFR mutations and has been associated with premature cardiovascular disease [112,113,114]. The link between homocysteine and cardiovascular disease can be explained by several different mechanisms, such as endothelial dysfunction, oxidative damage, increased collagen synthesis and increased vascular smooth muscle cell proliferation [112,115,116,117]. Recently, cross-sectional studies have looked for an association between the increase in plasma homocysteine level and the presence of structural and functional alterations of the arterial wall in asymptomatic subjects with cardiovascular pathologies [118]. From a functional point of view, elevated plasma homocysteine has been associated with impaired endothelium-dependent vasodilatation. From a structural point of view, it was highlighted that the increase in homocysteinemia would be associated with the restoration of the carotid artery independently of the known determining factors, of arterial diameter and thickness, and suggests that HHCY could equally constitute a marker of atherosclerosis. It has been shown that folate supplementation would allow control of moderate HHCY.

A direct causal relationship between HHCY induction and accelerated atherosclerosis has been reported in apolipoprotein E (apoE)-deficient mice [119]. It has been reported that the myocardium is particularly vulnerable to damage by HHCY, which is associated with the production of reactive oxygen species and causes the progression of cardiovascular disease [120]. The correlation between HHCY and atherosclerosis comes from the comparative analysis of the clinical and biological profiles described for the three enzyme deficiencies of genetic origin, associated with severe HHCY in humans. The pro-atherogenic role of HHCY has been confirmed by clinical and epidemiological studies. Most studies have reported a strong association between HHCY and total occlusive arterial disease, ischemic heart disease, and peripheral arterial disease. In parallel, the total of prospective studies performed in patients affected by declared cardiovascular diseases demonstrated that homocysteinemia is correlated with the occurrence of cardiovascular accidents. However, prospective studies have provided conflicting results, some of them considering HHCY to be causal in the clinical manifestations of atherosclerosis, and others noting the absence of a causal relationship.

If these different opinions do not allow us to state that HHCY is an independent cardiovascular risk factor, they prove that HHCY actively contributes to the development of cardiovascular diseases in high-risk patients.

Ponti et al. [121] reported that they started a prospective study of 500 patients to evaluate the predictive value of homocysteine as a specific marker for cardiovascular risk in patients with COVID-19, grouped into “usual,” “sub intensive,” and “intensive” [121].

## 4. Homocysteine: A Closer Look at the Correlation with SARS-CoV-2 Infection 

To date, there are few studies on the involvement of homocysteine in the COVID-19 disease. Table 2 schematically presents the data published in the literature regarding plasma homocysteine levels and SARS-CoV-2 infection.

The first prospective single-center cohort study on this subject is presented by Yang et al. [122]. The authors investigated different parameters such as homocysteine and predictors of imaging progression on lung computed tomography scans in the case of patients infected with SARS-CoV-2. Their results indicate that homocysteine levels were correlated with the severity of lung lesions (assessed by chest CT scans) [122]. Until now, few studies have been presented in the literature regarding the correlation of homocysteine with pulmonary imaging progression in patients with SARS-CoV-2 infection. Ponti et al., [123] in a study conducted on Italian-nationality patients hospitalized with COVID-19 investigated the role of homocysteine in this disease. In agreement with the obtained results, they propose plasma HCY levels and MTHFR gene sequencing as markers for the clinical management of SARS-CoV-2 infection [123]. Although several studies report homocysteine as a strong predictive marker for the severity of SARS-CoV-2 infection, the results of the study by Khalid et al. [128] reported that it is a moderate predictive marker for the disease.

The renin-angiotensin-aldosterone system is central to circulatory blood volume and peripheral vascular resistance regulation; its main components are ACE, angiotensin II/angiotensin II receptors, types AT1 and AT2. ACE’s main role is converting angiotensin I to angiotensin II which binds to type I Ang II receptors thus triggering an intracellular pathway leading to a plethora of potentially detrimental vascular effects (sodium and water retention, vasoconstriction, inflammation, oxidative stress, apoptosis) [131,132]. Conversely, the ACE2 enzyme is involved in angiotensin II proteolysis; the resulting Ang (1–7) peptide coupled to Mas receptors has opposite biological effects to angiotensin II. SARS-CoV and SARS-CoV-2 spike proteins have an affinity for ACE2 which acts as a receptor and mediates membrane fusion and cell entry [133,134]. Cell entry, aside from viral ACE2 binding, alters the balance between angiotensin II and Ang (1–7) effects leading to pro-inflammatory events and triggering cytokine cascade activation [135,136]. A possible correlation between HHCY and SARS-CoV-2 infection may be explained by the direct involvement of homocysteine angiotensin II type I receptor activation, contributing to the severity of vascular/endothelial lesions [137]. There are multiple mechanisms accounting for AT1 activation—homocysteine-triggered upregulation of AT1 transcription has been described but also direct interaction between homocysteine and the AT1 receptor with intracellular conformational changes and subsequent activation [137]. There are three biological active forms of homocysteine (free, oxidized and reduced) [138,139,140]. There are no clear data on the differences between them in terms of intensity of interaction with AT1 and cardiovascular effects. Considering these interactions, it would be useful to find out if patients with comorbidities associated with high plasma homocysteine levels (such as megaloblastic anemia) have worse SARS-CoV-2 infection outcomes. Should this be the case therapeutic approaches may be developed although reducing homocysteine plasma levels by vitamin B supplementation seemed not to alleviate cardiovascular risk in renal transplant patients [141]. 

Another possible correlation between HHCY and the severity of SARS-CoV-2 infection may be related to the presence of the C677T point mutation in the gene encoding MTHFR [142]. Herrera et al. [143] performed a retrospective study on 334 patients having had coronary, pulmonary, or other location arterial thrombosis to investigate the relationship between the C677T polymorphism, homocysteine concentration and prothrombotic biomarkers. Their results indicate the correlation of HHCY in the presence of the T allele in the C677T gene with pulmonary embolism and acute myocardial ischemia [143]. The study by Ponti et al. [144] showed, in the Latino population, a strong correlation between the C677 T variant and death due to SARS-CoV-2 infection.

## 5. Conclusions

Although it is known that SARS-CoV-2 infection causes severe coagulation disorders and that these play a crucial role in the evolution of patients, currently many aspects of the underlying pathophysiology remain unclear. Homocysteine has been proposed as a marker of cardiovascular risk and complications in hospitalized patients with SARS-CoV-2 infection. Its inclusion in laboratory markers may be of particular importance to predict disease severity. 

## Figures and Tables

**Table 1:** Causes of elevated homocysteine.

Factors	Comments	References
Genetic abnormalities		
MTHFR deficiency	Homozygous with thermolabile C677T mutation NM_005957.4(MTHFR): c.665C > T (p. Ala222Val) tend to have higher homocysteine levels.	[57,58,59]
MTHFR defect	Homozygous thermostable mutations, rare in population.	[46,60,61,62]
CBS deficiency	Heterozygosity led to higher homocysteine levels following methionine loading.	[63,64,65]
CBS defect	Homozygotes (rare) tend to develop severe basal hyperhomocysteinemia.	[46,60]
Methionine synthase deficiency	Caused by biallelic defects in the MTR (5-Methyltetrahydrofolate-Homocysteine Methyltransferase) gene is a rare inborn error of metabolism affecting the homocysteine remethylation pathway, leading to intermediate or severe hyperhomocysteinemia.	[45,66,67,68]
Nutritional factors		
Vitamin B6 deficiency	Contribute to impaired transsulfuration and an abnormal methionine load test, which is associated with premature vascular disease.	[69,70]
Vitamin B_12_ deficiency	Serum total homocysteine concentrations are markedly increased in most patients.	[69,71,72]
Folate deficiency	Folate acts as a donor of methyl groups for the homocysteine and deficiency can cause moderate/intermediate hyperhomocysteinemia.	[46,73]
Medications		
Methotrexate	Inhibits dihydrofolate reductase.	[46,74]
Metformin	Inhibit the absorption of Vitamin B12.	[74,75]
Oral contraceptive pills	Mechanism for how oral contraceptive pills increase the level of homocysteine is no known. The proposed mechanisms are: (a) synthesis of free radicals inside the body that directly increase homocysteine concentration and, (b) decreases of the bioavailability of cofactors utilized to degrade homocysteine.	[46,76]
Colchicine	Inhibit Vitamin B12 absorption.	[77,78]
Methylprednisolone	Reduces the concentration of Vitamin B6.	[74]

**Table 2:** Studies indicating a correlation between HCY levels and COVID-19 patients.

Reference	Participants	Results
Yang et al., 2020 [122]	273 patients with COVID-19	Homocysteine was reported as predictive marker for imaging lung progression in patients with SARS-CoV-2 infection.
Ponti et al., 2021 [123]	304 patients hospitalized for COVID-19	The authors report that homocysteine is a predictive marker for the outcome of patients infected with SARS-CoV-2.
Smirnova et al., 2021 [124]	104 patients with COVID-19	Patients with COVID-19 presented a hypercoagulable state and homocysteine can be a diagnostic indicator for the outcome of the disease.
Ali et al., 2021 [125]	42 participants with COVD19, survival 19 patients and non-survival 23 patients	One week after starting medication, homocysteine concentration increased in the non-survival group and decreased in the survival group.
Petelina et al., 2021 [126]	65 patients after COVID-19-associated pneumonia	Elevated levels of homocysteine three months after COVID-19, endothelial dysfunction and thrombophilia are indicators of prolonged arterial inflammatory syndrome.
Fouda et al., 2022 [127]	80 participants, 40 children with COVID-19 and 40 healthy as control	In patients with COVID-19, the level of homocysteine showed a significant increase with an average value of 27.5 μmol/L, which suggests that it could be a marker for predicting the severity of the disease.
Khalid et al., 2022 [128]	90 participants, 45 hospitalized patients with COVID-19, and 45 healthy as control	Moderate correlation between hyperhomocysteinemia and the severity of SARS-CoV-2 infection.
Khidoyatovna et al., 2022 [129]	80 patients with COVID19	Homocysteine was significantly higher in patients with the non-wild-type allele of the MTHFR gene polymorphisms 677 C > T and 1298 A > C compared to the control group.
Keskin et al., 2022 [130]	151 participants, 117 patients with COVID-19 and 34 healthy as control	Hyperhomocysteinemia can be considered a risk factor in patients with COVID-19 and can predict the severity of the disease.

## Data Availability

Not applicable.
